# Evaluation of a Triple-Helical Peptide with Quenched Fluorophores for Optical Imaging of MMP-2 and MMP-9 Proteolytic Activity

**DOI:** 10.3390/molecules19068571

**Published:** 2014-06-23

**Authors:** Xuan Zhang, Jamee Bresee, Philip P. Cheney, Baogang Xu, Manishabrata Bhowmick, Mare Cudic, Gregg B. Fields, Wilson Barry Edwards

**Affiliations:** 1 Department of Radiology, University of Pittsburgh, Pittsburgh, PA 15219, USA; E-Mails: xuanzh2001@hotmail.com (X.Z.); breseeje@upmc.edu (J.B.); 2 Department of Chemistry and Biochemistry, University of Denver, Denver, CO 80208, USA; E-Mail: pcheney@du.edu; 3 Department of Radiology, Washington University in St Louis, St Louis, MO 63110, USA; E-Mail: baogangx@mir.wustl.edu; 4 Torrey Pines Institute for Molecular Studies, 11350 SW Village Parkway, Port St. Lucie, FL 34987, USA; E-Mails: mbhowmick@tpims.org (M.B.); mcudic@tpims.org (M.C.); gfields@tpims.org (G.B.F.)

**Keywords:** matrix metalloproteinase-2 (MMP-2), MMP-9, gelatinase, triple-helical peptides, optical imaging, fluorescence molecular tomography (FMT)

## Abstract

Matrix metalloproteinases (MMP) 2 and 9, the gelatinases, have consistently been associated with tumor progression. The development of gelatinase-specific probes will be critical for identifying *in vivo* gelatinoic activity to understand the molecular role of the gelatinases in tumor development. Recently, a self-assembling homotrimeric triple-helical peptide (THP), incorporating a sequence from type V collagen, with high substrate specificity to the gelatinases has been developed. To determine whether this THP would be suitable for imaging protease activity, 5-carboxyfluorescein (5FAM) was conjugated, resulting in 5FAM_3_-THP and 5FAM_6_-THP, which were quenched up to 50%. 5FAM_6_-THP hydrolysis by MMP-2 and MMP-9 displayed k_cat_/K_M_ values of 1.5 × 10^4^ and 5.4 × 10^3^ M^−1^ s^−1^, respectively. Additionally 5FAM_6_-THP visualized gelatinase activity in gelatinase positive HT-1080 cells, but not in gelatinase negative MCF-7 cells. Furthermore, the fluorescence in the HT-1080 cells was greatly attenuated by the addition of a MMP-2 and MMP-9 inhibitor, SB-3CT, indicating that the observed fluorescence release was mediated by gelatinase proteolysis and not non-specific proteolysis of the THPs. These results demonstrate that THPs fully substituted with fluorophores maintain their substrate specificity to the gelatinases in human cancer cells and may be useful in *in vivo* molecular imaging of gelatinase activity.

## 1. Introduction

Matrix metalloproteinases (MMPs) are a family of zinc dependent endopeptidases that are capable of degrading extracellular matrix (ECM) components such as collagen, elastin, gelatin, and proteoglycans. The MMPs can be classified into different groups. One such group is the gelatinases, which consists of the type IV collagenases MMP-2 and MMP-9. Normally, MMP levels are low; however, they are elevated during cell proliferation, cell migration, and tissue remodeling events [1]. MMPs are initially synthesized as inactive zymogens (pro-MMP) and are secreted into the extracellular space, anchored to the cell membrane, or remain localized inside of the cells [2,3]. Upon cleavage of the pro-peptide domain, the active site is exposed within the catalytic domain [4]. MMPs may also be activated under oxidative stress by the combined action of peroxynitrite and glutathione [5]. MMPs, including the gelatinases have been implicated in various disease processes, such as cancer. MMP-2 and MMP-9 activity is upregulated in breast [6,7], colorectal [8,9], prostate [10,11], and gastric cancers [12,13], fueling the development and metastatic processes of these tumors through degradation of ECM, regulation of angiogenesis, stromal invasion [14], and extravasation at the metastatic site [15].

Molecular optical imaging has become of great interest recently for its use in non-invasive diagnostics and intraoperative fluorescence-guided surgery [16,17,18,19]. The development of optical imaging probes capable of detecting gelatinase activity would be useful in these *in vivo* applications since gelatinase activity has good prognostic capability. For instance, MMP-2 and -9 activity have been hypothesized to be involved in metastatic events and predict patient outcome in breast [20,21,22], prostate [23,24], and colorectal cancers [8,9].

Previously, our group and others have synthesized peptide substrates for detecting gelatinase activity [25,26,27,28,29,30]. To our knowledge, our triple-helical peptide (THP) has shown some of the best specificity for the gelatinases over other MMP family members. Selectivity towards the gelatinases is of high significance since other MMP family members, such as MMP-1, MMP-3, MMP-13, and MMP-14 are often expressed by the cancer cells of the primary tumor, surrounding stromal cells, and endothelial cells [18]. This THP is a self-assembling homotrimeric THP that includes the native collagen type V sequence, GPPG~VVGEKGEQ (the scissile bond lies between G and V), in the single-stranded peptides. Inclusion of repeating Gly-Pro-4-hydroxy-L-proline (GPO) triplets at both the N- and C-termini allow for the self-assembly of three single-stranded peptides into a single THP, imparting specificity for cleavage by the gelatinases in addition to the native collagen sequence [31]. While this peptide did not discriminate between the gelatinases, this is not a serious shortcoming since the gelatinases are secreted and activated simultaneously [32,33].

After the success of our previous studies, we continue to further evaluate the potential of triple-helical peptides for proteolytic imaging [34]. The goal of this study was to determine whether single-stranded peptides bearing 5-carboxyfluorescein (5FAM) dyes would assemble into a triple-helix and function as a substrate for gelatinases with kinetic parameters suitable for the *in vivo* detection of gelatinase activity. 5FAM was chosen due to its commercial availability, amenability to solid phase peptide synthesis, high quantum yield, and the ubiquity of fluorescein filter sets in a variety of fluorescence imaging equipment [35]. Moreover, the choice of a single dye for homo-quenching simplifies the synthesis of the probe. The on-resin conjugation procedure of 5FAM to single-stranded peptides was modified to increase conjugation efficiency. The resulting triple-helical peptides (5FAM-THPs) bear fluorophores conjugated to ε-amino Lys groups that flank the hydrolysis site. The kinetic parameters and structural properties of 5FAM-THPs were evaluated, as well as whether 5FAM-THPs bearing homodimeric dyes would visualize gelatinase activity secreted by human cancer cells with confocal fluorescence microscopy.

## 2. Results and Discussion

### 2.1. 5FAM-THP Design and Synthesis

**Figure 1:**
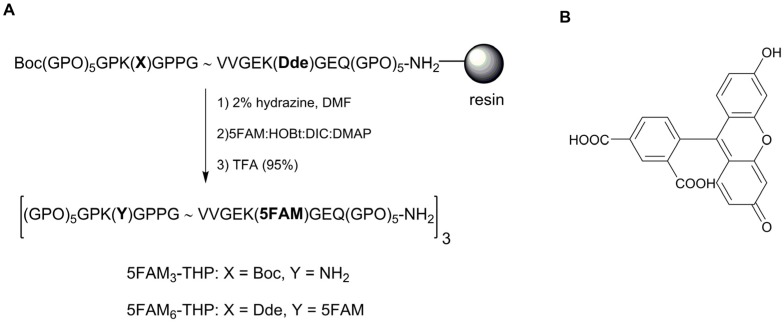
(**A**) Solid phase synthesis of 5FAM_3_-THP and 5FAM_6_-THP (“O” represents 4-hydroxyproline). The tilde represents the site of hydrolysis by gelatinases (**B**) 5-carboxyfluorescein (5FAM).

Single-stranded collagen peptides were synthesized via solid phase peptide synthesis with a pair of Lys residues flanking the hydrolysis site to provide ε-amino groups for conjugation of either one or two 5FAM fluorescent dyes per single-stranded peptide (Figure 1). After cleavage from resin, three single-stranded collagen peptides labeled with 5-carboxyfluorescein (5FAM) self-assembled into the THPs, 5FAM_3_-THP and 5FAM_6_-THP. Mass spectrometric analysis of the purified 5FAM-THPs indicated mono- or di-substituted single-stranded peptides for 5FAM_3_-THP and 5FAM_6_-THP, respectively: 5FAM_3_-THP: calculated (M+H)^+^ = 4,463, observed (M+H)^2+^ = 2,233, (M+H)^3+^ = 1,489, (M+H)^4+^ = 1,117; 5FAM_6_-THP: calculated (M+H)^+^ = 4,819, observed (M+H)^+^ = 4,823, (M+2Na)^+^ = 4,845.

The design of 5FAM-THP was based on the previous design of a fluorogenic THP substrate that fluoresced in the visible region (fTHP). The mechanism of quenching for fTHP was between 7-methoxycoumarin and 2,4-dinitrophenyl groups which were conjugated to the ε-amino groups of Lys [29]. For synthetic ease, here we have chosen the fluorescein analogue 5FAM to serve as the fluorescent dye to report gelatinase activity since it is readily available from a variety of commercial sources, exhibits a high quantum yield, and is highly amenable to solid-phase peptide synthesis. A previously reported method, which detailed optimized coupling conditions with DIC, HOBt, and 5FAM to achieve complete coupling of 5FAM to the N-terminal amino group, did not yield complete coupling with our single-stranded collagen peptide [35]. The addition of 4-dimethylaminopyridine (DMAP) was necessary to result in complete coupling of 5FAM to the Lys ε-amino groups, which was confirmed by both a ninhydrin test and ESI-TOF mass spectrometry. Previous work has shown that during coupling of activated 5FAM to resin bound peptides, more than one 5FAM molecule was conjugated via ester formation between the carboxylic acid and the phenolic oxygen of 5FAM [35]. Treatment with piperidine was required to remove these adducts. Here, DMAP may function as a nucleophile to remove the undesired esters during coupling of the 5FAMs. Alternatively, DMAP may act as an acyl transfer-reagent and facilitate the reaction between the Lys ε-amino groups and 5FAM carboxy groups, making the unwanted attack of the 5FAM phenolic oxygen that resulted in ester bond formation less favored [36,37]. When DMAP was not present in the coupling cocktail, the amide bond formation was too slow (Lys ε-amino groups are not as reactive as the N-terminal amino groups) and the side-reaction (esterification) occurred. This confirmed a new approach to coupling 5FAM to resin bound peptides with high coupling efficiency and obviated the need for an extra treatment with the nucleophilic base piperidine.

### 2.2. 5FAM-THP Quenching

**Figure 2:**
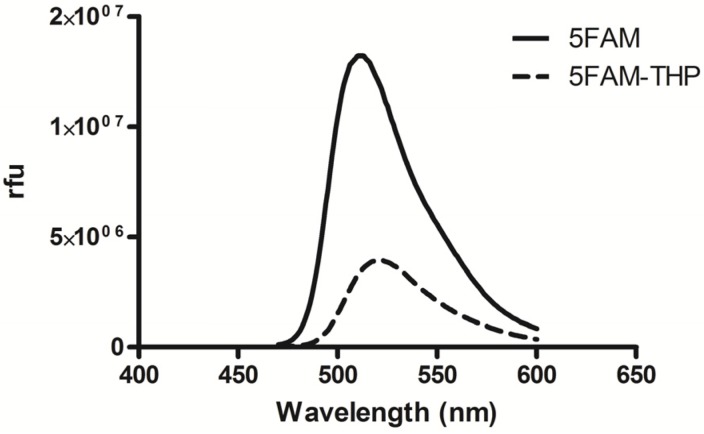
Fluorescence emission spectra of 5FAM (solid) and 5FAM_6_-THP (dashed), where λ_excitation_ = 480 nm. The decrease in fluorescence signal for 5FAM_6_-THP demonstrates the quenching of fluorescence of the fluorogenic peptide due to close proximity of the 5FAM fluorophores.

A fully substituted 5FAM-THP was desired in anticipation of maximized self-quenching of the fluorophores upon THP self-assembly. The Kaiser test showed the completed substitution of 5FAM on the ε-amino groups of Lys of both conjugates while the peptide was resin bound and verified a substitution level of three 5FAM: one THP for 5FAM_3_-THP and six 5FAM: one THP for 5FAM_6_-THP. By comparing the fluorescence of a fully proteolyzed sample of 5FAM-THP with an undigested sample, an average value of quenching of 32% ± 0.5% for 5FAM_3_-THP and 50% ± 2% for 5FAM_6_-THP of 5FAM fluorescence was observed in the THPs. The fluorescence emission spectra of the 5FAM-THPs showed a slight shift to the red relative to unconjugated 5FAM with no other perturbations, except a decrease in fluorescence, which was due to the close proximity of the fluorophores (Figure 2). We previously showed that for a THP substituted with 1.8 5FAM per six available ε-amino groups, there was 38% quenching [34]. In this study, similar quenching levels were observed with 5FAM_3_-THP. However, complete substitution of the ε-amino groups in the 5FAM_6_-THP conjugate resulted in 50% quenching of the fluorophores, which translated to a 2-fold increase in fluorescence after complete proteolysis.

To calculate the homo-FRET efficiency, the distance between the α-carbons of the lysines that are nine residues apart, which approximates the distance between dye-conjugated lysines that flank the hydrolysis site, were measured from a model of a THP [38]. Additional measurements were made between the α-carbons of inter-chain Lys residues. These distances ranged from 26 to 32 Å. Utilizing an average distance between α-carbons (R = 30 Å,), a Forster distance was estimated (R_0_ = 44 Å). Thus, the homo-FRET efficiency, when the fluorophores are separated by distance of 26 to 32 Å, ranges from 63% to 58%. These efficiencies are in good agreement with the measured level of quenching which was 50%. The level of quenching was related to the number of 5FAM substitutions, which might suggest that a FRET mechanism in the same peptide chain played an important role here. In contrast, previous work with more flexible single-stranded peptides, such as the MMP-7 substrate GVPLSLTMGC bearing a heterologous donor and acceptor pair of near infrared dyes on the N- and C- termini, yielded a level of 85% quenching with an approximate 7-fold increase in fluorescence after digestion with MMP-7 [39]. In flexible peptides, the mechanism of quenching has been attributed to the formation of intramolecular dimers between the donor and quencher fluorophores [40]. The triple-helical conformation appears to be too rigid to permit extensive intramolecular association of the 5FAM fluorophores and this may have limited the extent of quenching. Further, this level of quenching for 5FAM-THPs was lower than that observed with other fluorogenic probes that have been used *in vivo*, where the level of quenching has varied from 88% to 96% [30,33,41]. While the level of quenching of our 5FAM-THPs is sufficient for *in vitro* work, a higher level of quenching will most likely be desired for *in vivo* studies; however, it is important to note that *in vivo* imaging of proteolytic activity is in a nascent stage and values for minimum amounts of quenching have not yet been established.

### 2.3. 5FAM-THP Kinetics

**Figure 3:**
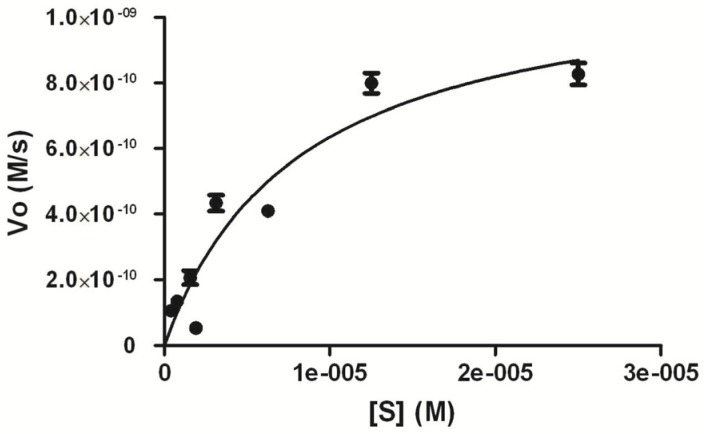
Representative Lineweaver-Burk analysis of MMP-2 and MMP-9 mediated proteolysis of 5FAM_6_-THP. Enzyme kinetics were conducted by incubating activated MMP-2 or -9 enzyme with varying concentrations (1-100 µM) of THP substrate. Initial velocities were obtained from plots of fluorescence with respect to time using only the linear portion of the data. Relative fluorescence units were converted from known concentrations of 5FAM in assay buffer. K_M_ values were determined by non-linear regression and fit with GraphPad Prism. Error bars are contained within the symbols.

In addition to achieving a high level of quenching, a fluorogenic peptide should be efficiently hydrolyzed by the enzyme. 5FAM-THPs were efficiently hydrolyzed by MMP-2 and MMP-9, indicating that the addition of the fluorophores did not perturb the triple-helical structure or change the substrate avidity of the THPs for the gelatinases (Table 1, Figure 3). The k_cat_/K_M_ values for human-MMP-2 hydrolysis of 5FAM_3_-THP and 5FAM_6_-THP were 1.1 × 10^4^ and 1.5 × 10^4^ M^−1^ s^−1^, respectively while the k_cat_/K_M_ values for human-MMP-9 hydrolysis of 5FAM_3_-THP and 5FAM_6_-THP were 4.6 × 10^3^ and 5.4 × 10^3^ M^−1^ s^−1^, respectively, with similar values reported for hydrolysis of the 5FAM-THPs with rat-MMP-2 and -9.

**Table 1:** Enzyme kinetic parameters for gelatinase mediated hydrolysis of 5FAM-THPs.

Substrate	K_M_ (μM)	k_cat_ (s^−1^)	k_cat_/K_M_ (M^−1^ s^−1^)	MMPs
fTHP *^a^*	4.4	0.061	14,002	MMP-2
fTHP *^a^*	8.1	0.044	5,400	MMP-9
5FAM_3_-THP	8.9 ± 0.7 *^b^*	0.092	10,337	MMP-2
5FAM_3_-THP	10.2 ± 0.4 *^b^*	0.047	4,628	MMP-9
5FAM_3_-THP	3.3 ± 0.3 *^b^*	0.051	15,421	MMP-2 (Rat)
5FAM_3_-THP	8.9 ± 3.3 *^b^*	0.040	4,507	MMP-9 (Rat)
5FAM_6_-THP	4.3 ± 0.3 *^b^*	0.065	15,116	MMP-2
5FAM_6_-THP	6.6 ± 0.2 *^b^*	0.036	5,400	MMP-9

*^a^* fTHP sequence is (GPhP)_5_GPK(Mca)GPPG~VVGEK(Dnp)GEN(GPhP)_5_-NH_2,_ where Mca is (7-methoxycoumarin-4-yl)acetyl and Dnp is 2,4-dinitrophenyl; *^b^* error values are ± standard error of the mean (*n* = 3).

**Figure 4:**
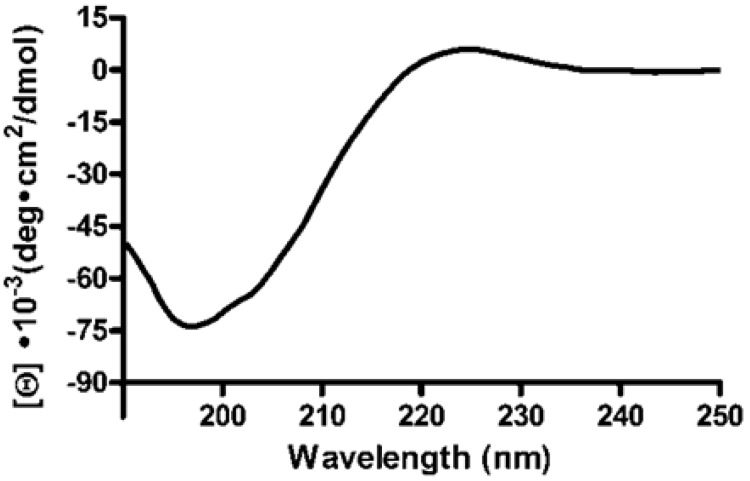
Circular dichroism spectra of 5FAM_6_-THP (3.45 µM, 0.1% aqueous acetic acid). Spectra were collected over λ = 190–300 nm at 10 °C.

**Figure 5:**
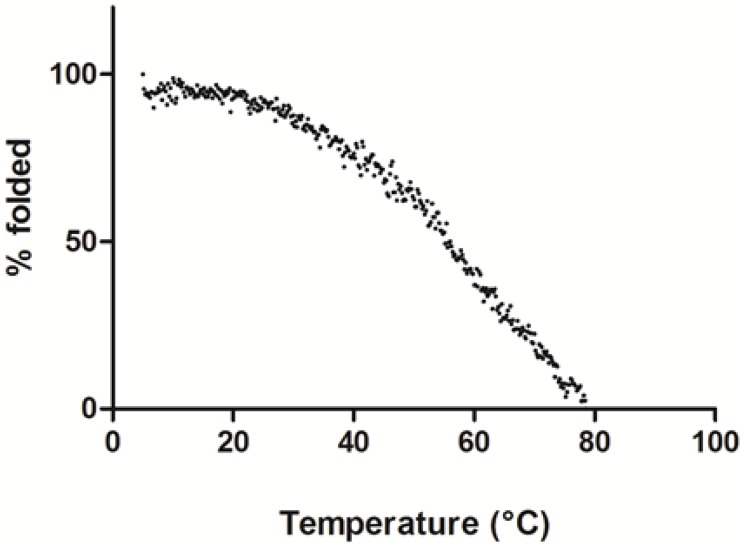
Thermal transition curves for 5FAM_6_-THP determined by monitoring molar ellipticity (Θ = 225 nm) while the temperature was increased.

Other examples of protease imaging probes suggest that there is a range of k_cat_/K_M _values of ~1 × 10^4^ to 7 × 10^6^ M^−1^ s^−1^ for proteolytic substrates that successfully imaged *in vivo* proteolytic activity [32,42,43]. The k_cat_/K_M _value for 5FAM-THP hydrolysis by MMP-2 falls inside this range, suggesting that THPs bearing quenched homodimeric dyes would be effective for gelatinase imaging *in vivo* providing that ample quenching is achieved. Since the K_M_ and k_cat_ values between the THPs were similar, the addition of the relatively bulky 5FAM fluorophores did not have a great effect on the 5FAM-THP’s substrate avidity for the gelatinases (Table 1). Moreover, the circular dichroism spectra were strongly indicative of the triple-helical conformation for both 5FAM_3_-THP and 5FAM_6_-THP (Figure 4) [29,44]. More specifically, the maximum [Θ] value at λ ~222–225 nm and the minimum [Θ] value at λ ~190 nm (Figure 4) are hallmarks of triple-helical structure, as is a sigmoidal melting curve when examining [Θ] = 225 nm as a function of increasing temperature (Figure 5). The k_cat_/K_M_ value of a substrate is an important criterion as it provides a benchmark for the evaluation of a quenched substrate’s applicability for *in vivo* molecular imaging. Those substrates with the highest values would be expected to be the most rapidly hydrolyzed before wash-out from the target site, resulting in greater signal-to-noise. 5FAM_6_-THP was chosen to continue with further studies due to its higher level of quenching compared to conjugate 5FAM_3_-THP.

### 2.4. Microscopy of 5FAM-THP Hydrolysis and MMP Zymography

**Figure 6:**
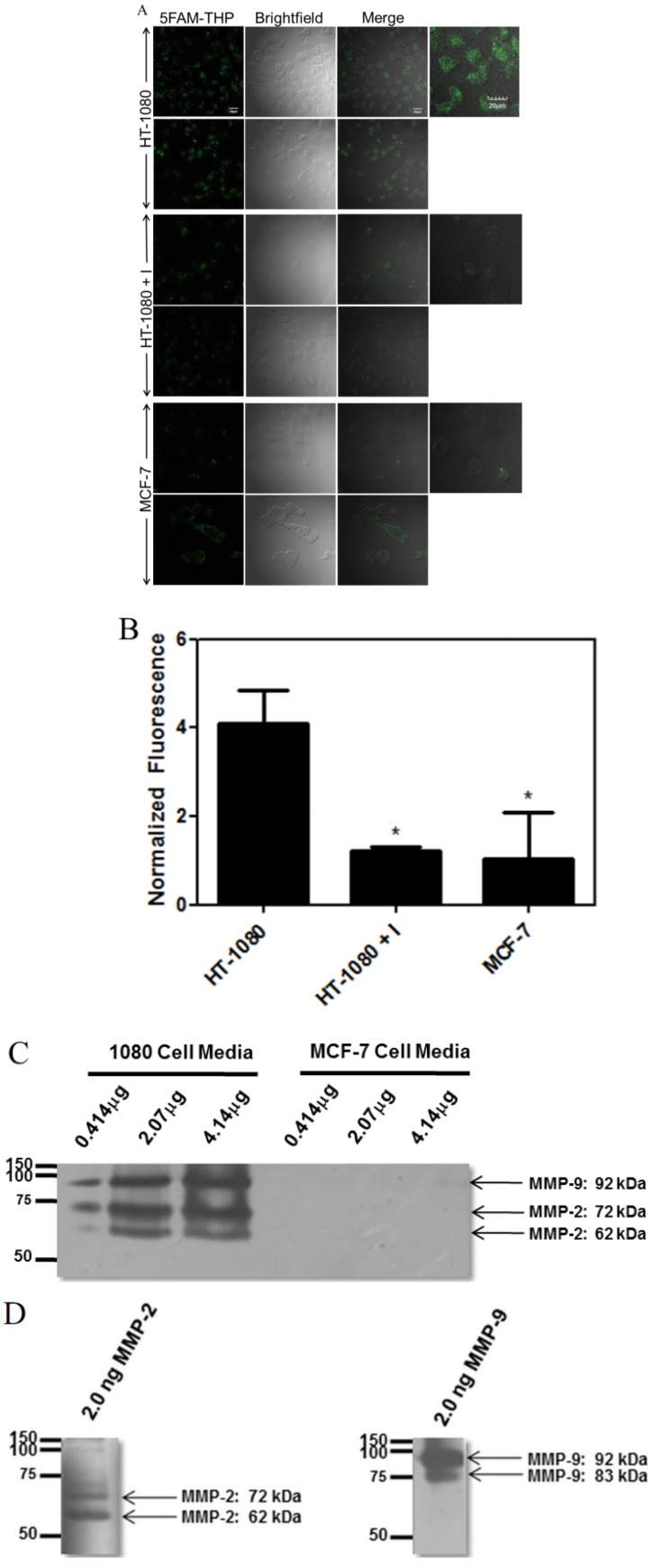
(**A**) Confocal fluorescence microscopy showing the hydrolysis of 5FAM_6_-THP by HT-1080 human fibrosarcoma cells. HT-1080 or MCF-7 human breast cancer cells were treated with 5FAM_6_-THP (1 µM) for 1 h. For inhibition studies, cells were pre-incubated with SB-3CT (1 µM) for 30’ prior to 5FAM_6_-THP incubation. Two image fields are shown per condition. The first column represents the fluorescence from 5FAM-THP. The second column represents the corresponding brightfield image. Overlays of the fluorescence and brightfield images are shown in the third column (taken using the 10× objective) with higher magnification images in the fourth column (taken using the 60× objective) to show cell internalization of 5FAM-THP. Scale bars are indicated in the first image of each column. (**B**) Mean cell associated fluorescence quantified with FV1000 software. (*n* = 10 cells, “I” denotes SB-3CT, “1080” denotes HT-1080 cells). P-values were determined using a Student’s two-tailed t-test; “*” denotes *p <* 0.05). (**C**) Zymogram depicting gelatinase content of cell-associated media with varying total protein loading. Varying levels of total protein from the conditioned media (0.41 µg, 2.07 µg, and 4.14 µg) were loaded into each well of a 10% Zymogram Gel with gelatin. Proteins were electrophoresed at 100 V for 90 min and after electrophoresis, the proteins were renatured in 2.5% Triton X-100. The gel was then incubated overnight at 37 °C in development solution and stained with Coomassie Blue. (**D**) Zymograms of MMP-2 and MMP-9 standards that were used in enzymology experiments and in Figure 6C to verify gelatinase content of cell-associated media.

Selective proteolysis of 5FAM-THPs by gelatinases is necessary for *in vivo* applications. Confocal fluorescence microscopy of cells incubated with 5FAM_6_-THP verified that 5FAM-THPs were capable of visualizing gelatinase activity in gelatinase positive HT-1080 human fibrosarcoma cells relative to gelatinase negative MCF-7 human breast adenocarcinoma cells, without non-specific fluorescence due to denaturation or non-gelatinoic proteolysis (Figure 6A). When 5FAM_6_-THP was incubated with HT-1080 cells, which secrete 72 kDa-MMP-2 and 94 kDa-MMP-9 in culture [33,45], intracellular fluorescence was observed, which appeared to be vesicularly concentrated in some cases, which is indicative of proteolysis by the gelatinases. In contrast when 5FAM_6_-THP was incubated with MCF-7 cells, which do not secrete 72 kDa-MMP-2 or 94 kDa-MMP-9 [46], low levels of fluorescence were observed and there was no internalization of fluorescence (Figure 6A). Addition of the gelatinase inhibitor SB-3CT greatly reduced fluorescence levels when 5FAM_6_-THP was incubated with HT-1080 cells by three-fold (Figure 6). This indicated that the observed fluorescence was due to gelatinase specific proteolysis of 5FAM_6_-THP and not due to proteolysis from other enzymes or other non-gelatinase specific processes (Figure 6A; the images shown are the center plane of the cells). Viability tests confirmed that cells used in the study were viable (≥90% viable; *n* = 2 field of views per condition) for the duration of the experiment. Gelatinase zymography confirmed the expected gelatinase production by HT-1080 and MCF-7 cells [5,47,48]. HT-1080 cells produced both MMP-2 (68 kDa and 72 kDa) and MMP-9 (92 kDa) and MCF-7 cells did not produce detectable amounts of the gelatinases (Figure 6C,D). Taken together, the observed intracellular fluorescence within HT-1080 cells was mediated by specific MMP-2 and MMP-9 hydrolysis of 5FAM_6_-THP and was neither due to non-specific processes nor due to a loss of cell viability.

The attenuation of the fluorescence signal in HT-1080 cells incubated with the gelatinase inhibitor SB-3CT was an indication that the observed fluorescence was, in part, due to gelatinase activity. The levels of SB3-CT were 72-fold the K_i_ value for MMP-2 (K_i_ = 0.0139 μM [49]) which ensures approximately 99% occupancy of MMP-2 at equilibrium. The inability to completely inhibit apparent proteolysis of 5FAM_6_-THP may have been due to non-equilibrium conditions resulting in uninhibited gelatinase activity or due to fluorescence from incomplete quenching of the 5FAM fluorescent dyes within 5FAM_6_-THP. This hypothesis is supported by the low levels of fluorescence observed in SB-3CT treated HT-1080 cells and in the gelatinase negative MCF-7 cells. The gelatinase mediated intracellular fluorescence in HT-1080 cells is of particular interest because it could influence net tumor-associated fluorescence *in vivo*.

Gelatinase mediated proteolysis of 5FAM_6_-THP resulted in a loss of the triple-helical conformation and a subsequent diffusion of the peptide fragments with concomitant increase in fluorescence as the proximity of the fluorophores decreased [50]. The single-stranded proteolytic fragments were then internalized into the HT-1080 cells, and in some cases, were intra-vesiculary located. MCF-7 cells, in contrast, did not exhibit observable intracellular fluorescence. Without gelatinase activity, 5FAM_6_-THP remained intact, with the 5FAM molecules quenched and without proteolytic fragments for cell internalization. While a complete study of the mechanism of internalization was beyond the scope of this investigation, it is useful to note that functionally active MMP-2 is non-covalently bound to the surface of numerous cancer cells. MMPs, including MMP-2, bind other ubiquitous receptors, such as αvβ3 integrin, which is generally ubiquitously expressed, albeit at various levels, in xenografted tumors and in human tumor tissue [51,52,53,54], and the cell surface proteoglycan CD44, which is overexpressed on many tumors and cancer stem cells. An example of these interactions is the interaction of the CD44 splice variant CD44v_3,8-10_ in metastatic breast cancer cells with active MMP-9 within invadopodia structures [55]. The interaction of CD44v_3-10_ and MMP-9 is also found in prostate cancer cells [56]. Association of CD44 with active MMP-9 in metastatic breast cancer and melanoma cells leads to the activation of TGF-β and the promotion of the degradation of type IV collagen with subsequent cell invasion [57,58]. The close association of the gelatinases with CD44 and αvβ3 may explain the strong intracellular signal. Mechanisms of internalization of the proteolyzed fragments may include passive egress of the proteolytic fragments into the cytosol as well as pinocytosis or receptor mediated endocytosis (in the case of αvβ3) into endosomes.

## 3. Experimental

### 3.1. Chemicals

All standard peptide synthesis chemicals were analytical reagent grade or better and purchased from EMD Biosciences (San Diego, CA, USA) or Fisher Scientific (Pittsburgh, PA, USA). 9-Fluorenylmethoxycarbonyl-amino acid derivatives were obtained from EMD Biosciences. Amino acids are of the L-configuration (except for Gly). 5FAM (5-carboxyfluorescein, single isomer) was from Invitrogen (Carslbad, CA, USA). The MMP-2 and MMP-9 inhibitor, SB-3CT, was from Enzo Life Sciences (Plymouth Meeting, PA, USA).

### 3.2. Solid Phase Peptide Synthesis

The THPs were synthesized as previously described [59]. Either one or two 5FAMs per single-stranded peptide were conjugated to the peptides depending on the choices of the protecting groups on one of ε-Lys groups. The generic synthetic route is shown in Figure 1. After removal of the Dde protecting groups (2% hydrazine, DMF), rinsing (3 × 5 mL DMF, 10% aqueous DMF, CH_2_Cl_2_, CH_3_OH), and drying (*in vacuo*), 5FAM was conjugated to the ε-amino groups of Lys (resin:5FAM:HOBt:DIC:DMAP = 1:X:X:2X:X, X = 3 or 6) in DMF. After reaction (16 h), the mixture was filtered, and rinsed (3 × 5 mL DMF, CH_2_Cl_2_, CH_3_OH). The Kaiser test was negative indicating complete coupling of 5FAM. The peptide was cleaved from the resin (water-TFA 1:19, 3 h), diluted with water, and lyophilized. The crude mixture was re-dissolved (0.1% acetic acid) and purified by size exclusion gel chromatography (G-25). Substitution levels were determined by absorption (480 nm) utilizing the ninhydrin method and bovine insulin as a standard [41] and molecular weight was confirmed by ESI-TOF-MS.

### 3.3. THP Quenching

To determine whether conjugation of 5FAM to the peptide changed the emission spectra, the fluorescence of 5FAM-THPs were compared to that of 5FAM free in solution. The concentrations of both 5FAM and 5FAM_6_-THP were adjusted to 2 µM (±0.05 µM, PBS). Emission spectra (λ_em_ = 400 to 600 nm, λ_ex_ = 480) were obtained. To determine the amount of quenching, the fluorescence of unhydrolyzed 5FAM-THPs (f*_intact_*) (5FAM_3_-THP and 5FAM_6_-THP) were compared with the fluorescence of completely hydrolyzed 5FAM-THPs (f*_hydrolyzed_*), utilizing [(f*_hydrolyzed_* − f*_intact_*)/f*_hydrolyzed_*] × 100 = percent quenched. Three concentrations of 5FAM-THPs (0.5 µM, 0.15 µM, and 0.07 µM, *n* = 3 per sample) were digested with MMP-9 (14 nM) until fluorescence reached a plateau with no further increases (2.5 h).

### 3.4. Calculation of Förster Distance

To calculate the Förster distance, a model of a collagenous generic THP was used. This model, consisting of repeating GAA (alanine residues were substituted in place of proline and hydroxyproline for simplification) triplets of 30 amino acids ([(GAA)_10_]_3_) was composed from the high resolution crystal structures of several THPs in order to predict peptide backbone and α-carbon locations within the helix [38]. A PDB format file (GAA_THim.pdb) with the coordinates of a model THP was imported in to Chemdraw3d Pro 9.0 to make the measurements. Utilizing the software’s measurement function, the distances between α-carbons of A18 and A27 of one chain of the THP were determined. These two amino acids correspond to the pair of lysine residues (that were conjugated to the dye) that flank the hydrolysis site. The distance between the α-carbon of A18 in the first chain and that of the α-carbons of the other two alanines (A27) from the other two chains were also measured. 

### 3.5. Enzyme Assays

MMP-2 (72 kDa) and MMP-9 (94 kDa) for the enzyme assays were purchased from Enzo Life Sciences. 72 kDa MMP-2 (rat) and 94 kDa MMP-9 (rat) were from R & D Biosystems (Minneapolis, MN, USA). The 72 kDa MMP-2 (or MMP-2) was activated using 4-aminophenylmercuric acetate (APMA). An APMA solution was made at 10 mM (3.5 mg/mL) in assay buffer (50 mM tricine pH 7.4, 50 mM NaCl, 10 mM CaCl_2_, 0.05% Brij-35) and diluted to 2 mM prior to addition to the proenzyme solution. APMA (2 mM) was added to the 72 kDa MMP-2 solution in equal parts by volume for a final solution of 1 mM. This solution was allowed to sit for 1 h at 37 °C before dilution in assay buffer. The 94 kDa MMP-9 (or MMP-9) was activated by the addition of TPCK-trypsin (0.50 mg/mL) in 20 µL of activation buffer (50 mM Tris, pH 7.5, 200 mM NaCl, 5 mM CaCl_2_, 0.02% Brij-35). This solution was allowed to sit at 37 °C for 20 min, after which 2 µL of aprotinin (1.0 mg/mL) was added. The resulting solution was brought up to a volume of 522 µL.

Enzyme kinetics were carried out using activated 72 kDa MMP-2 and 94 kDa MMP-9 at nominal concentrations of 10 or 20 nM. The amount of active enzyme (E_t_) was taken from published values of the percent active enzyme generated under identical conditions (70%, MMP-2, 100% MMP-9), or the values provided by manufacturer [60,61]. Reactions were performed in triplicate in 96-well microtiter plates in a total volume of 50 or 200 µL. Data was collected using a Synergy HT microplate reader (Bio-Tek, Winooski, VT, USA). The K_M_ values of 5FAM-THP were determined by incubating the substrate (1 to 100 µM) with activated 72 kDa MMP-2 and 94 kDa MMP-9 in assay buffer. The fluorescence released by enzyme-mediated hydrolysis was measured at multiple time points on a microtiter plate reader. Initial velocities were obtained from plots of fluorescence with respect to time using only the linear portion of the data. Relative fluorescence units were converted from known concentrations of 5FAM in assay buffer. K_M_ values were determined by non-linear regression and error values are expressed as the 95% confidence interval (Graphpad Prism, version 5.1, San Diego, CA, USA). k_cat_ was determined from E_t_ and K_M_. The kinetics data are presented as Lineweaver-Burke plots.

### 3.6. Circular Dichroism Spectroscopy and Melting Curves

Circular dichroism spectra were collected over a range of λ = 190–300 nm on a JASCO J-815 instrument (Tokyo, Japan) using a 1 mm path length quartz cell at 10 °C. Temperature control was maintained with a Peltier equipped temperature control unit.

### 3.7. MMP Zymography

The cell growth media of HT-1080 and MCF-7 cells (DEME, 10% fetal calf serum, 100 units/mL penicillin) was replaced with serum free medium to generate conditioned media 24 h prior to zymography. The protein content of the conditioned media from each cell line was analyzed and subjected to zymographic analysis. Varying levels of total protein from the conditioned media (0.41 µg, 2.07 µg, and 4.14 µg) were loaded into each well of a 10% Zymogram Gel with gelatin (BioRad, Hercules, CA, USA). Proteins were electrophoresed at 100 V for 90 min in Tris-glycine buffer containing 0.1% SDS. After electrophoresis, the proteins were renatured in 2.5% Triton X-100 after which the gel was incubated overnight at 37 °C in development solution (50 mM Tris, 200 mM NaCl, 5 mM CaCl_2_, 0.02% Brij-35). The gel was stained for 1 h at room temperature in 40% methanol, 10% acetic acid, 0.05% Coomassie Blue R-250, followed by destaining in 40% methanol, 10% acetic acid.

### 3.8. Confocal Microscopy

Cells were grown on Lab-Tek slides in DMEM, 10% fetal calf serum, 100 units/mL penicillin. The medium was replaced and cells were incubated with 5FAM_6_-THP (1 μM, 1 h). For the inhibitor studies, cells were pre-treated with a MMP-2 and MMP-9 inhibitor (SB-3CT, 1 μM, 30 min) after which 5FAM_6_-THP (1 μM, 1 h) was added to the wells so that the inhibitor was present for the entire incubation. The concentration of 5FAM_6_-THP was chosen to ensure significant saturation of the gelatinases to produce a detectable signal that would rise above level of autofluorescence in a reasonable amount of time before cell viability was lost. After rinsing, a cell viability assay was performed with ethidium homodimer-1 (EthD-1; 1 μM, 1 h). Then the slides were rinsed with PBS, mounted, and coverslipped. They were visualized using an Olympus FV1000 microscope (Center Valley, PA, USA) using a 20X/0.95 W water immersion objective. Fluorescence emission of 5FAM_6_-THPwas detected from 490 to 550 nm using a 488 nm laser at 5% power. Images of each group of slides were acquired with the same microscope settings during a single imaging session, allowing for quantitative analysis of cellular fluorescence. Relative fluorescence of the cells was quantified with FV1000 software in terms of fluorescence per unit area. Relative fluorescence of ten cells of each image was determined and the results averaged (with standard deviation; *n* = 10). The results were analyzed with an unpaired, two-tailed t-test (GraphPad Prism version 4.00 Software).

## 4. Conclusions

The goal of the study was to synthesize and evaluate homotrimeric triple-helical peptides composed of single-stranded peptides bearing 5FAM fluorescent dyes flanking the hydrolysis site for their ability to detect gelatinase activity in cell culture. These 5FAM-THPs were characterized for the ability of the single-stranded peptides, bearing one or two fluorescent dyes, to self-assemble into a triple-helix and be efficiently hydrolyzed by the gelatinases. The 5FAM fluorophore was added while the single stranded-peptide was resin bound and the addition of DMAP greatly increased the coupling efficiency. After release from the resin and purification, mass spectrometry data and the strong positive and negative molar ellipticities of 5FAM-THPs indicated that the single-stranded peptides bearing either one of two 5FAMs had indeed self-assembled into a triple-helix. While the level of quenching, up to 50%, was lower than that of other proteolytic sensors that have been used *in vivo* in the past, it should be noted that minimum quenching levels for *in vivo* fluorescence imaging of proteolytic activity with quenched probes are not known. Additionally, the kinetics of enzymatic hydrolysis of 5FAM-THPs (MMP-2 hydrolysis of 5FAM_6_-THP k_cat_/k_M_ was 1.5 × 10^4^ M^−1^ s^−1^) were well within the range of kinetic parameters of enzymatic probes that have successfully visualized proteolytic activity *in vivo.* Finally, the gelatinases secreted from HT-1090 human cancer cells were capable of proteolyzing 5FAM_6_-THP, which was confirmed by an increase in fluorescence signal resulting from THP hydrolysis and dequenching of 5FAM fluorophores. This proteolysis was blocked by a gelatinase inhibitor. Fusrthermore, 5FAM_6_-THP hydrolysis did not occur in gelatinase negative MCF-7 cells, indicating that 5FAM-THP proteolysis was gelatinase specific and did not result from THP denaturation or non-specific proteolysis. Taken together, these data support the use of THPs labeled with fluorophores for *in vivo* imaging of gelatinase activity.
